# Pushing the Boundaries of Molecular Property Prediction for Drug Discovery with Multitask Learning BERT Enhanced by SMILES Enumeration

**DOI:** 10.34133/research.0004

**Published:** 2022-12-15

**Authors:** Xiao-Chen Zhang, Cheng-Kun Wu, Jia-Cai Yi, Xiang-Xiang Zeng, Can-Qun Yang, Ai-Ping Lu, Ting-Jun Hou, Dong-Sheng Cao

**Affiliations:** ^1^ Xiangya School of Pharmaceutical Sciences, Central South University, Changsha 410013, Hunan, P. R. China.; ^2^ Shangqiu Normal University, School of Information Technology, Shangqiu 476000, Henan, P. R. China.; ^3^ College of Computer, National University of Defense Technology, Changsha 410005, Hunan, P. R. China.; ^4^ Department of Computer Science, Hunan University, Changsha 410082, Hunan, P. R. China.; ^5^ Institute for Advancing Translational Medicine in Bone and Joint Diseases, School of Chinese Medicine, Hong Kong Baptist University, Hong Kong SAR 999077, P. R. China.; ^6^ Innovation Institute for Artificial Intelligence in Medicine of Zhejiang University, College of Pharmaceutical Sciences, Zhejiang University, Hangzhou 310058, Zhejiang, P. R. China.

## Abstract

Accurate prediction of pharmacological properties of small molecules is becoming increasingly important in drug discovery. Traditional feature-engineering approaches heavily rely on handcrafted descriptors and/or fingerprints, which need extensive human expert knowledge. With the rapid progress of artificial intelligence technology, data-driven deep learning methods have shown unparalleled advantages over feature-engineering-based methods. However, existing deep learning methods usually suffer from the scarcity of labeled data and the inability to share information between different tasks when applied to predicting molecular properties, thus resulting in poor generalization capability. Here, we proposed a novel multitask learning BERT (Bidirectional Encoder Representations from Transformer) framework, named MTL-BERT, which leverages large-scale pre-training, multitask learning, and SMILES (simplified molecular input line entry specification) enumeration to alleviate the data scarcity problem. MTL-BERT first exploits a large amount of unlabeled data through self-supervised pretraining to mine the rich contextual information in SMILES strings and then fine-tunes the pretrained model for multiple downstream tasks simultaneously by leveraging their shared information. Meanwhile, SMILES enumeration is used as a data enhancement strategy during the pretraining, fine-tuning, and test phases to substantially increase data diversity and help to learn the key relevant patterns from complex SMILES strings. The experimental results showed that the pretrained MTL-BERT model with few additional fine-tuning can achieve much better performance than the state-of-the-art methods on most of the 60 practical molecular datasets. Additionally, the MTL-BERT model leverages attention mechanisms to focus on SMILES character features essential to target properties for model interpretability.

## Introduction

Though biotechnology has made significant advances, drug development is still a long, expensive, and complex process, which usually takes 10 to 15 years and billions of dollars [1]. There are several critical bottlenecks in this process, such as finding out validated molecular targets, designing and/or discovering promising lead compounds, optimizing pharmacokinetics and toxicity, and evaluating efficacy and clinical safety [2,3]. In these scenarios, the application of computational methods, particularly molecular property prediction methods, can alleviate the excessive dependence on time-consuming and labor-intensive experiments [4,5]. Therefore, the development of computational methods to predict various properties of chemical compounds has attracted extensive attention in the last few decades [6,7].

For molecular property prediction, one of the primary challenges is to learn expressive representations of molecular structures. Traditional molecular representation methods, which rely on sophisticated handcrafted features such as molecular fingerprints and/or descriptors, frequently suffer from lengthy design procedures and limited adaptability [8–10]. In recent years, the surging deep learning (DL) methods deliver a data-driven way to automatically learn molecular representations from primary data in the end-to-end training [11]. DL approaches have replaced the conventional paradigm of machine learning (ML) based on expert knowledge, enabling superior generalization ability and versatility in a wide variety of domains, such as molecular generation [12], drug-target affinity prediction [13], and protein structure prediction [14]. Inspired by the success of DL, several studies have been performed to apply well-established DL models to low-level molecular representations, including molecular graphs and SMILES (simplified molecular input line entry specification) [15–17].

Molecular graphs are natural representations for chemical molecules, which consist of atoms (nodes) connected through chemical bonds (edges) and are hence ideally suited for graph neural network (GNN). Up to now, various GNN architectures have been successfully applied to the prediction of molecular properties [11,18]. However, limited by overfitting and oversmoothing problems, existing GNNs are typically too shallow (generally 2 to 3 layers), thus limiting their ability to extract deep-level patterns. The SMILES strings encode the composition and connection information of molecules in ASCII (American Standard Code for Information Interchange) characters and are widely used in cheminformatics to store molecular structures [19]. Various DL models suitable for text processing, such as long- and short-term memory [16,20], convolutional neural networks [21,22], and Transformers [15], have been used to extract features from SMILES strings for molecular property prediction and achieved superior speed and performance with sufficient training data.

Generally, DL models require a large amount of labeled data to achieve high effectiveness and good generalization ability. For example, in image classification tasks, people usually collect millions of images to train their DL models [23]. Unfortunately, for most bioactivity-related tasks, labeled data are quite limited because of expensive and time-consuming laboratory experiments [24]. The scarcity of training data greatly increases the overfitting risk and reduces the generalization abilities of data-hungry DL approaches [25].

A viable solution to the data scarcity problem is to harness information and knowledge contained in other labeled tasks or even unlabeled data [26]. Several biochemical data, such as absorption, distribution, metabolism, excretion, and toxicity (ADMET) properties, are highly interrelated [27]. As an example, lipophilicity is often related to many ADMET properties. Thus, multitask learning can be used to exploit the correlation across different molecular property tasks and increase the model performance. While the use of multitask learning has been investigated for molecular property prediction, there are some limitations including possible performance degradation for unrelated tasks due to low model capacity and a lack of interpretability [2,27]. Except for learning representation from labeled data, unlabeled data contain rich knowledge and subtle patterns that are of high importance to representation learning and can be exploited through unsupervised learning [28,29]. In recent years, unsupervised learning methods have been widely used and have posed a considerable impact on computer vision and natural language processing [30,31], such as SimCLR [30] and BERT (Bidirectional Encoder Representations from Transformer) [32]. Inspired by these successes, unsupervised learning has been employed for molecular property prediction [33–35]. One strategy is to construct auto-encoder models that transform discrete SMILES representations to and from multidimensional continuous representations, which can then be used for downstream prediction tasks [33]. However, the SMILES recovery-based molecular representations may not be quite optimal for general prediction tasks and cannot be further optimized. Inspired by the success of the BERT model in natural language processing, a SMILES-BERT model was proposed by considering SMILES as sequences, in which a part of SMILES tokens is masked, and the BERT model learns to predict the missing part based on the available tokens [34]. In this process, the model is driven to mine the contextual information in SMILES strings. However, SMILES-BERT models are fine-tuned separately for each downstream task, which is quite inefficient when multiple properties need to be predicted and eliminates the opportunity to leverage the useful information contained in multiple related tasks to improve their generalization performance [34]. Furthermore, the SMILES-BERT model only uses the canonical form of SMILES strings to train their model, which limits its ability to capture the key relevant patterns in complex SMILES.

Another way to alleviate the data scarcity issue is data augmentation. As discussed in a previous study [16], a given molecule can be presented by different SMILES strings by varying starting atoms and traversal orders. Thus, each training sample can be expanded by a different number of SMILES representations to increase data diversity and help to learn the key relevant patterns hidden in the complex syntax of SMILES strings. Additionally, the SMILES enumeration can also be used in the test phase to correct potential prediction biases and therefore make a robust and accurate prediction. Concretely, in the test phase, we can generate multiple SMILES strings for a given test sample and perform a fusion operation on all the predictions of these SMILES strings to obtain the final prediction result.

In this study, we attempted to develop a novel multitask learning framework called MTL-BERT to alleviate the data scarcity problem by combining large-scale unsupervised learning, multitask learning, and SMILES enumeration together. The proposed MTL-BERT model is firstly pretrained on a large amount of unlabeled molecular data to mine the rich contextual information in the SMILES strings. In the fine-tuning stage, the pretrained model is trained on multiple tasks jointly to mine and share relevant information in similar tasks. The training data from multiple tasks can also serve as an inductive bias by imposing constraints on each other, thus boosting prediction accuracy and learning speed. Additionally, the SMILES enumeration continues to be utilized as a data augmentation strategy to substantially increase the data diversity in the pretraining, fine-tuning training, and test phases. By combining the 3 strategies together, MTL-BERT may provide a solution to molecular property prediction tasks with insufficient data. Experimental results demonstrate that the pretrained model can generate context-sensitive representations for SMILES tokens by investigating what the MTL-BERT model learned in unsupervised pretraining, and the ablation study proves that multitask learning and SMILES enumeration can indeed boost the model performance. Moreover, we conducted extensive experiments to evaluate the MTL-BERT method on a wide range of drug discovery-related tasks. The evaluation results show that the pretrained MTL-BERT model with a little extra fine-tuning can significantly outperform the state-of-the-art methods on a wide range of molecular property prediction datasets. In addition, the MTL-BERT model leverages attention mechanisms to focus on SMILES character tokens essential to the target property and provides valuable clues to analyze and optimize molecules. The superior performance, high execution efficiency, and excellent interpretability establish our proposed model as a competitive choice for molecular property prediction in drug discovery.

## Results

### Overall pipeline

**Fig. 1.**
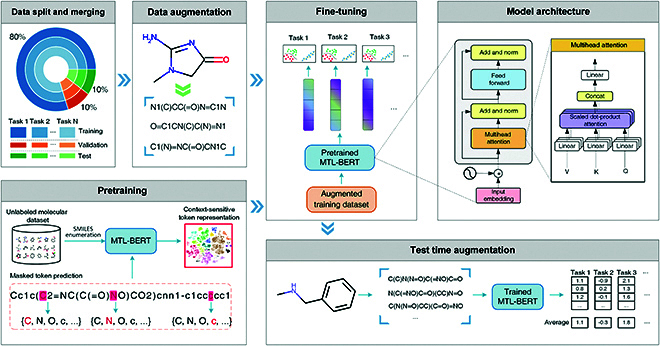
Pipeline overview of the MTL-BERT model.

The overview pipeline of MTL-BERT is shown in Fig. 1. The MTL-BERT model is firstly pretrained through the masked token prediction task on a large amount of unlabeled molecular data to mine contextual information in the SMILES strings. In the pretraining stage, the SMILES strings are firstly enumerated by using different starting atoms and traversal orders. Then, these SMILES strings are tokenized and further randomly masked for pretraining prediction. The SMILES augmentation strategy can substantially increase the data diversity and effectively learn the semantic information from the SMILES strings. Next, each dataset for the multiple molecular property prediction tasks is randomly split into the training, validation, and test datasets with a ratio of 8:1:1. After that, the training, validation, and test datasets are concatenated into multitask training styles. Then, these datasets are augmented 20 times by random SMILES enumeration. According to previous research on SMILES enumeration [16], increasing the data size by 20 times can effectively improve the performance of the model, but more augmentation times can only bring a very limited performance improvement and will greatly increase the computational load. To generate the unique SMILES strings for each molecule, if an enumeration is identical to previous ones, we will repeat the search for new SMILES forms until 100 times. Next, the multitask training data are utilized to fine-tune the pretrained model. In the prediction phase, we carry out a fusion operation of all predictions for the enumerated SMILES from the same molecule to obtain the final prediction.

### MTL-BERT structure study

To find what kind of MTL-BERT structure could perform better on molecular property prediction tasks, we designed and compared 3 structures of different sizes. The model parameters and performance of the 3 types of MTL-BERT structures are listed in the Table. The pretraining recovery accuracy and the averaged fine-tuning performance were used as the evaluation metrics. From the Table, one can clearly see that the small model is inferior to the other 2 models because of fewer parameters and limited capacity. The large MTL-BERT performed best on the pretraining recovery task but slightly worse than the medium MTL-BERT on the fine-tuning tasks, probably due to slight overfitting. Thus, we chose the medium MTL-BERT structure since it takes much less training cost and can achieve better prediction performance.

**Table.** Parameters and performances of the 3 structures of MTL-BERT

Name	Layers	Heads	Embedding size	FFN size	Recovery accuracy	Performance
MTL-BERT_SMALL_	4	4	128	512	0.931	0.826
MTL-BERT_MEDIUM_	8	8	256	1,024	0.962	**0.852** (*P* < 0.07)
MTL-BERT_LARGE_	12	12	576	2,304	**0.974** (*P* < 0.03)	0.848

MTL-BERT, multitask learning Bidirectional Encoder Representations from Transformer. FFN, feed forward network.

### Ablation study: The MTL-BERT architecture is indeed effective

**Fig. 2.**
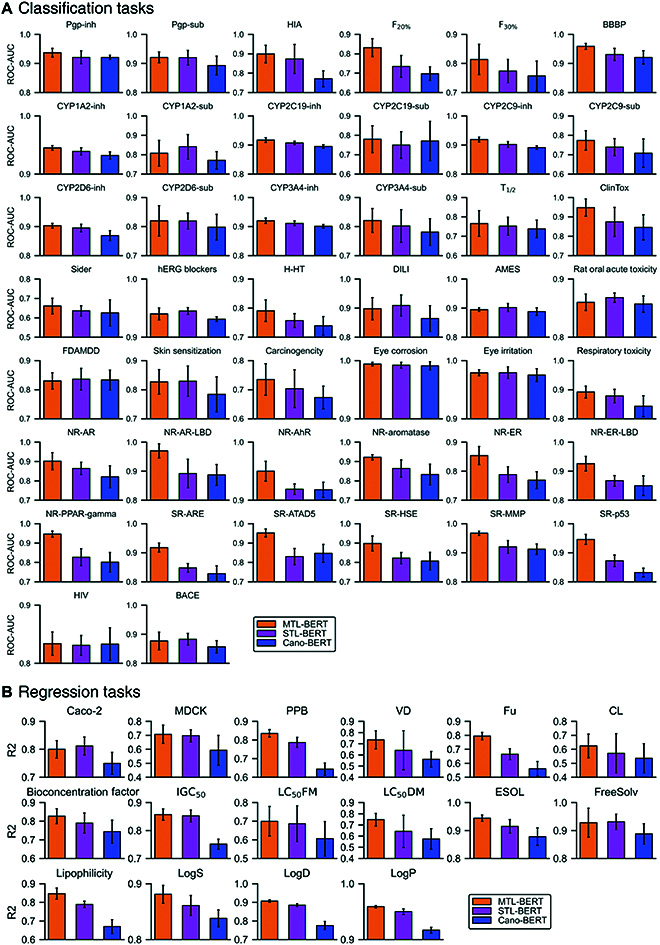
The performance comparison of MTL-BERT, STL-BERT, and Cano-BERT in the ablation studies for (A) classification tasks and (B) regression tasks.

To verify the effectiveness of the multitask learning and SMILES enumeration used in MTL-BERT, we performed an ablation study. We compared our developed MTL-BERT model with the single-task BERT (STL-BERT) model and the canonical SMILES-based BERT (Cano-BERT) model. STL-BERT applied the same settings as MTL-BERT but separately fine-tuned the pretrained model for each task. Cano-BERT removed the SMILES enumeration step in the MTL-BERT model, and only used the canonical SMILES to pretrain the BERT model and separately fine-tune the pretrained BERT model for each task. Herein, the developed Cano-BERT can be seen as the implementation of SMILES-BERT reported by Wang et al. [34]. The comparison results of the 3 different methods can be found in Fig. 2 and Tables S2 and S3. Compared with the STL-BERT model, the Cano-BERT model showed obvious performance loss in all tasks and even showed more than 10% degradation in some datasets such as CL (clearance of a drug), Fu (fraction unbound in plasma), and LC_50_DM [48-h *Daphnia magna* LC_50_ (concentration of the test chemical in water in mg/l that causes 50% of *Daphnia magna* to die after 48 h)]. These results demonstrate the necessity and effectiveness of the data augmentation strategy. SMILES enumeration in the pretraining and fine-tuning stages can greatly improve the data diversity and enable the model to find more truly relevant features to generate more expressive molecular representations. Furthermore, the SMILES enumeration during testing time can correct the prediction bias and provide a more robust prediction. SMILES enumeration in the 3 stages together greatly boosts the performance of the regression tasks by a nontrivial margin. Despite both models using SMILES enumeration, the MTL-BERT model outperformed the STL-BERT model on the majority of the datasets. For some datasets, such as F_20%_ (human oral bioavailability 20%), SR-ARE (antioxidant response element), and SR-ATAD5 (ATPase family AAA domain-containing protein 5), the MTL-BERT even improved by more than 5%. For several datasets such as AMES, CYP1A2-Sub, and FreeSolv, the MTL-BERT showed slightly worse performance where it fell short by less than 1.2%. These results prove that the multitask learning strategy can mine and share relevant information in similar tasks and therefore improve overall generalization capabilities. Except for better performance, the multitask learning strategy can greatly reduce memory usage and running times, proving that the MTL-BERT model has great practical value. In short, these results fully demonstrate the effectiveness of the multitasking and data augmentation strategies.

**Fig. 3.**
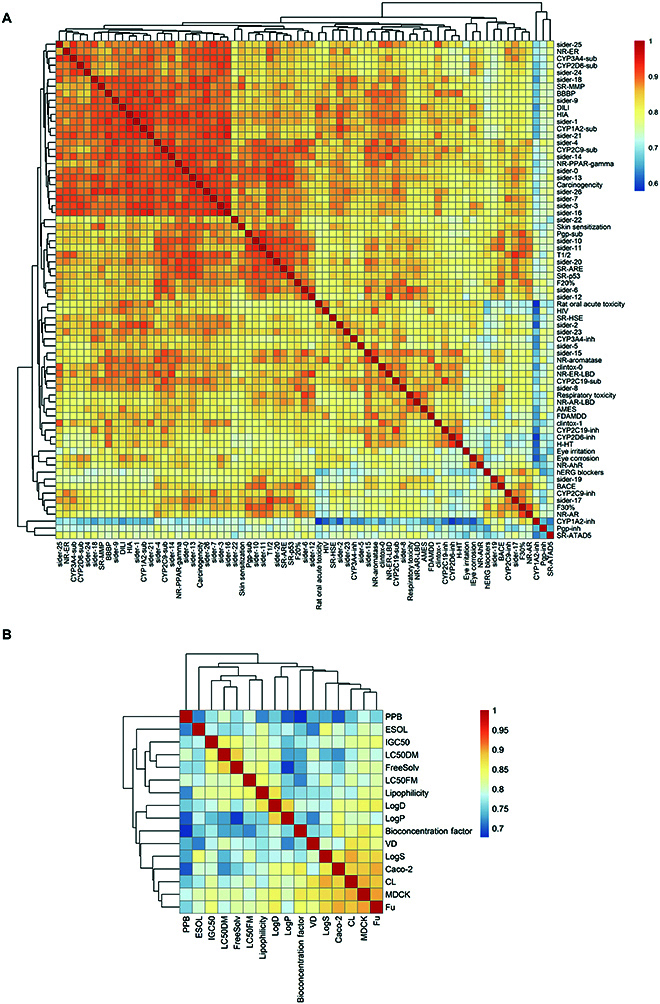
Correlation analysis between different task representations between (A) classification tasks and (B) regression tasks.

In addition, the MTL-BERT model uses attention weights to extract information from each token of the molecule to compose task-relevant molecular representations. Thus, we convert task-related weights into vectors and calculate the average correlation between these weight vectors. The correlation results between different tasks and the results of the hierarchical clustering according to these correlation coefficients are shown in Fig. 3. The hierarchical clustering reveals several clustering groups, suggesting that the MTL-BERT model can explore the correlations between different tasks. For example, the tasks LogD and LogP are clustered together, which may be due to the fact that both are related to water solubility.

### Comparison with other machine learning methods

We selected 5 state-of-the-art molecular property prediction models as the baselines for comprehensively evaluating our proposed MTL-BERT model. The first is the XGBoost model based on the Extended-Connectivity Fingerprints with diameter 4 (ECFP4-XGBoost) [8,9], which is a classical paradigm for molecular property prediction tasks. Moreover, 3 representative and widely used GNNs are also included as the baselines: graph attention network [36], graph convolutional network [37], and AttentiveFP [17]. The last method is based on the continuous-and-data-driven descriptor (CDDD) [33], and it consists of a fixed RNN (recurrent neural network) encoder that has been pretrained on a large number of unlabeled SMILES strings together with a fully connected neural network. For each task, we used the grid search algorithm to find the optimal hyperparameter settings for the baseline models.

**Fig. 4.**
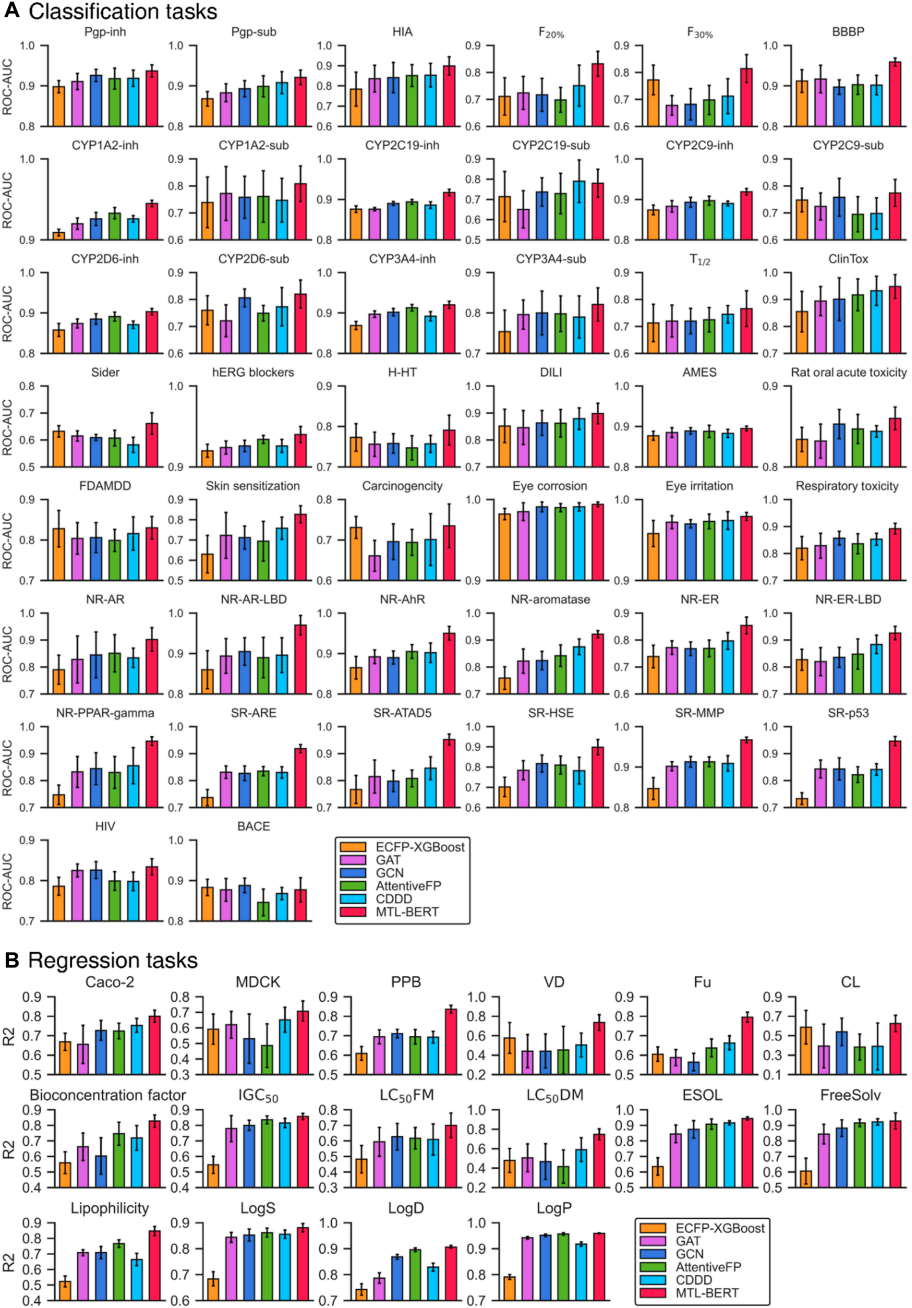
The performance comparison of the MTL-BERT and state-of-the-art models for (A) classification tasks and (B) regression tasks.

The prediction results are shown in Fig. 4 and Tables S4 and S5. Clearly, the ECFP4-XGBoost models perform poorly on most tasks, but well on some tasks, such as F_30%_ (human oral bioavailability 30%), BACE, and CL, showing very obvious performance differences. The reason could be that ECFP4 is a fixed-length molecular representation, and the information it contains may not be useful for a specific task. As molecular graphs contain more information than molecular fingerprints, 3 GNNs (i.e., graph attention network, graph convolutional network, and AttentiveFP) perform better than molecular fingerprints on most tasks. However, due to the scarcity of labeled data, these GNNs also show poor performance on some tasks. For example, on F_30%_, Carcinogenicity, CL, and VD, both GNN models fall behind the ECFP-XGBoost by more than 3%. The CDDD model, which trained an auto-encoder model on large-scale data, can capture molecular features in a data-driven way. The CDDD representation has made some progress in many tasks. However, it cannot be further optimized for specific tasks such as molecular fingerprints. Therefore, the information it contains may also not be sufficient for certain tasks, thus resulting in performance degradation in some tasks. The MTL-BERT model not only can learn rich molecular contextual information through pretraining but also can be optimized for specific problems. Combining the benefits of information sharing between multiple tasks and SMILES enumeration, the MTL-BERT model has made an advance on most tasks. Clearly, MTL-BERT performs better than all baseline models, except for 2 subtasks: CYP2C19-sub and BACE, where it fell short by less than 1.1%. The performance of MTL-BERT in NR-ER (estrogen receptor), NR-PPAR (peroxisome proliferator-activated receptor)-gamma, SR-ARE, SR-ATAD5, SR-HSE (heat shock factor response element), SR_MMP (mitochondrial membrane potential), Bioconcentration Factor, Fu, LC50FM, and lipophilicity tasks surpasses other models by more than 5%, and its performance in CL, PPB (plasma protein binding), VD (volume distribution), and LC_50_DM surpasses other models by more than 10%. The improvements of our model relative to the baselines on most datasets are statistically significant (95% confidence interval) according to the paired *t* test (*P* ≤ 0.001). These results convincingly highlight the excellent effectiveness and good generalizability of the proposed MTL-BERT model. In addition to better prediction performance, the MTL-BERT model does not need to perform a complex hyperparameter search for each task, and it runs very efficiently, which fully proves its potential as a good choice for molecular property predictions.

### Analysis of the representations of the SMILES tokens from the pretrained MTL-BERT model by t-SNE

**Fig. 5.**
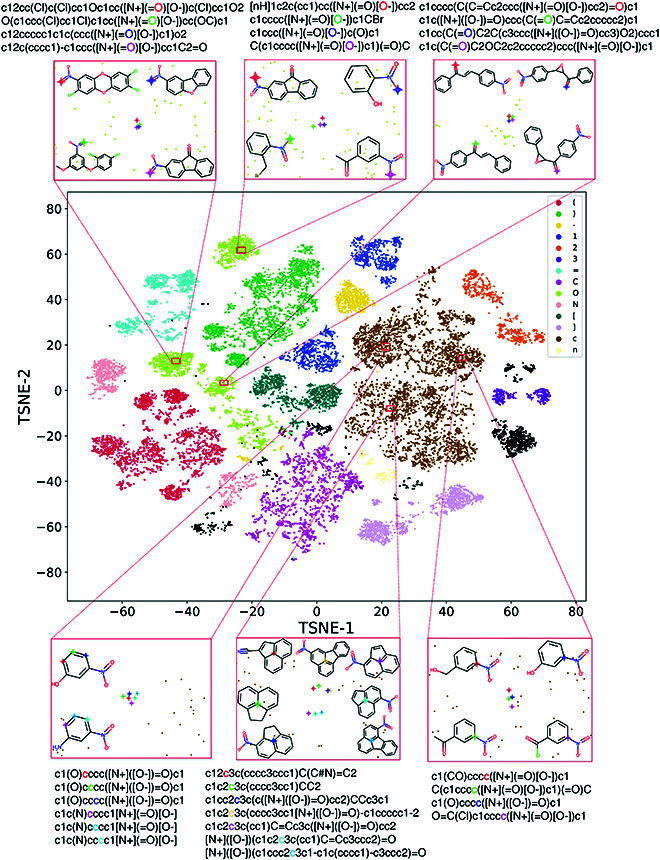
The t-SNE plot of token embedding vectors is colored by token category. In these enlarged graphs, the SMILES token, their corresponding embedding position, and their corresponding atoms in the molecule are marked with the same color.

To analyze what the MTL-BERT model learned in the pretraining stage, we visualized the representations of the SMILES string tokens generated by the pretrained MTL-BERT model and tried to find some interesting patterns hidden in the SMILES string space. Specifically, 1,000 molecules (including approximately 35,000 tokens) were randomly selected and fed into the pretrained MTL-BERT model without masking, and the output of the Transformer encoder layer was gathered to perform visualization analysis. In this way, a 256-dimensional vector was generated for each atom, and about 35,000 vectors were obtained in total. The classical dimensionality reduction method t-SNE (T-distributed stochastic neighbor embedding) [38] was used to visualize these high-dimensional vectors. As shown in Fig. 5, the tokens of different types are clearly clustered together and can be easily distinguished, demonstrating that the generated representations contain information of atomic types. Further observation shows that the atoms of the same type can be divided into several different groups. It seems that the generated atomic representations contain richer information than simple atomic types. To further observe the detailed patterns, we made some local enlarged graphs of token “O” and token “c”. In these enlarged graphs, we marked several token representations clustered in a small region and displayed the corresponding molecules. “O” tokens on the first and second enlarged graphs all represent the oxygen atoms from the nitrate group attached to the benzene rings, but the oxygen atoms on different graphs are in different positions. In the third enlarged graph, the “O” symbols represent the oxygen atoms in the carbonyl group attached to the benzene ring. These results clearly indicate that molecular neighborhood environments of tokens from nearby locations are similar, whereas those from distant locations are significantly different. The other 3 enlarged graphs show similar results and can further prove this finding.

The above results demonstrated that the generated token representations can successfully capture the contextual information in the SMILES strings. In this way, token representations can be regarded as continuous substructure representations, which should be extremely useful for downstream tasks.

### Analysis of MTL-BERT's attention

MTL-BERT utilizes attention mechanisms to aggregate information from all SMILES tokens to construct task-relevant molecular representations. Accordingly, the attention weights represent, in some sense, each SMILES token's specific contribution to the final molecular representation and could be considered as a relevant measure to the target property. Therefore, MTL-BERT provides a natural method for discovering the relationship between molecular substructures and molecular properties, which is critical for analyzing and optimizing molecules.

**Fig. 6.**
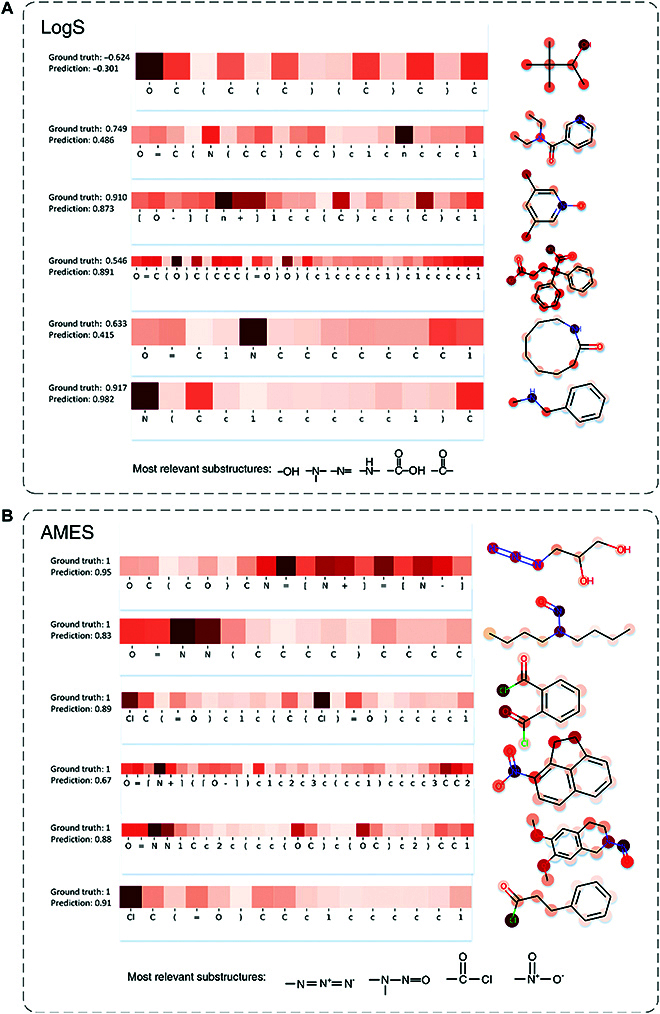
Visualization of the MTL-BERT's attention weights allocated to the SMILES tokens for the (A) LogD task and (B) AMES task. Darker colors indicate higher attention weights.

To verify whether the MTL-BERT model can reasonably assign the attention weights, we analyzed some molecules in the test sets of the LogS and AMES tasks. The LogS task is related to the water solubility of molecules. In Fig. 6A, it appears that more attention is being paid to polar groups, which are important factors in determining the solubility of molecules in water. The AMES task is related to the mutagenicity of molecules. According to Fig. 6B, the attention is mainly focused on the azide, nitrosamide, acylchloride, and nitrite groups, which have been shown to be mutagenic structural alerts due to their high appearance in mutagens [39]. The results show that MTL-BERT is capable of assigning attention weights to specific tasks, which is very important for medicinal chemists to explore the relationship between molecular properties and substructures to some extent.

## Discussion

For new drug development, accurate and efficient molecular property prediction is essential, as they can reduce reliance on time-consuming and labor-intensive experiments. Recently, data-driven DL models have shown great potential in molecular property predictions and have gained increasing attention. Despite their exceptional learning abilities, DL models need a large amount of labeled data in order to highlight relevant patterns and guarantee high performance. Unfortunately, the labeled data for the majority of bioactivity-related tasks are usually quite limited due to expensive and time-consuming laboratory experiments. Therefore, when applied to bioactivity-related tasks, DL models are quite prone to overfitting, thus resulting in poor generalizability.

In our solution, we jointly leveraged large-scale pretraining to learn from unlabeled data, multitask-based transfer learning to learn from other tasks, and data augmentation to increase the labeled data diversity. The combination of 3 strategies can make full use of available data information and alleviate the problem of data scarcity as much as possible. Large-scale pretraining can effectively dig out the rich knowledge and complex patterns hidden in unlabeled molecular data. By pretraining with masked token prediction tasks, we found that the proposed model can generate contextually sensitive representations of SMILES tokens, which is quite useful for downstream tasks. Since molecular properties are usually highly related, multitask learning can then be used to explore the similarities between different tasks to promote the overall performance by transfer learning. In addition, multitask learning can also greatly improve the prediction performance and reduce the computational cost by training multiple tasks at once. The SMILES enumeration, as an effective data augmentation strategy, can significantly increase the training data diversity and help to focus on more essential features, which are both used in pretraining and fine-tuning to extract key relevant features from SMILES strings. Furthermore, SMILES enumeration can also be used in the testing phase to correct possible model bias and to perform a more robust prediction. Experimental results demonstrate that our proposed strategies are very effective and can greatly improve prediction performance. In addition, the MTL-BERT model has certain interpretability due to its use of attention mechanisms, making it a helpful tool for medicinal chemists to explore the relationship between substructures and property endpoints of interest. With superior performance, high efficiency, and good interpretability, the MTL-BERT model provides a competitive choice for accurately and efficiently predicting molecular properties in drug discovery. More broadly, the paradigm of MTL-BERT can also be employed in other fields such as drug–protein interaction prediction, drug–drug interaction prediction, and so on. Specifically, in these fields, the proposed model can be used to learn a quite expressive representation of molecules according to limited labeled data.

## Materials and Methods

### Dataset collection

The training procedure of the MTL-BERT model consists of 2 stages: pretraining and fine-tuning. During pretraining, 1.7 million unlabeled molecules collected from the ChEMBL database [40] were used as the pretraining data to learn the contextual information in the SMILES strings. We randomly kept 10% of the entire pretraining data to evaluate the pretraining model. During fine-tuning, the pretrained model was further trained for the molecular property prediction tasks. Sixty datasets (16 for regression and 44 for classification) covering critical ADMET endpoints and various common molecular properties were collected from ADMETlab [41] and MoleculeNet [42] to train and evaluate MTL-BERT. The detailed information about these 60 datasets is listed in Table S1. All the molecules in these datasets are stored in the SMILES string format. Each dataset was randomly split into the training, validation, and test datasets with a ratio of 8:1:1. Each dataset covers one molecular property prediction task, except for ClinTox and SIDER, which have 2 and 27 tasks, respectively. Therefore, we ended up with 71 binary classification tasks and 16 regression tasks.

### Model architecture

As shown in Fig. 2, the MTL-BERT model utilizes the Transformer encoder part as a feature extractor, which takes advantage of multihead self-attention to capture both long-term and short-term dependencies, and different attention heads to focus on different aspects of patterns. The model architecture of MTL-BERT can be found in Fig. 1. Specifically, the MTL-BERT model consists of 3 components: an embedding layer, several self-attention layers, and a task-related output layer. In the embedding layer, the input tokens *x* = (*x*_1_, *x*_2_, …, *x*_*n*_) are mapped into learnable dense vectors *w* = (*w*_1_, *w*_2_, …, *w*_*n*_) through an embedding dictionary **D** ∈ R^V×F^, where *w*_*i*_ ∈ R^F^, V is the vocabulary size, and F is the embedding vector size. As the BERT model cannot automatically learn positional information, a predefined positional encoding vector needs to be added to each embedding vector in the embedding layer. In the multihead self-attention layer, the input data **Y** is mapped into H distinct query matrix $Q_{h}={\mathrm{YW}}_{h}^{Q}$, key matrix $K_{h}={\mathrm{YW}}_{h}^{K}$, and value matrix $V_{h}={\mathrm{YW}}_{h}^{V}$, respectively, where *h* = 1, 2, …, H, **Y** ∈ R^N×F^ is the input feature matrix, and $W_{h}^{Q}$, $W_{h}^{K}$, and $W_{h}^{V}$ are learnable parameters. After these linear projections, the scaled dot-product attention computes a sequence of outputs:

$$O_{h}=\text{softmax}\left(\frac{Q_{h}K_{h}^{T}}{\sqrt{d_{k}}}\right)V_{h}$$ (1)

where $\sqrt{d_{k}}$ is a scaling factor and **O**_*h*_ is the output of one head output of the attention layer.

Afterward, **O**_1_, **O**_2_,…, **O**_H_ are concatenated and linearly projected again to mix-up information of different heads. Upon the attention output, a position-wise feedforward network with 2 fully connected layers and a Gaussian Error Linear Units activation function [43] in the middle are stacked. Besides, all the self-attention sublayers and position-wise feedforward sublayers are followed by a normalization layer [44] and a residual connection to well increase the generalization ability and utilize the original information. More detailed settings can be found in the original literature [32,45].

The last layer is a fully connected neural network, which further processes the Transformer layer's output and performs specific classification or regression tasks. The embedding layer and the Transformer encoder layer are shared in the pretraining and fine-tuning stages. The last layer for the pretraining and fine-tuning stages is not shared and is called the pretraining head and the prediction head, respectively.

### Pretraining based on masked SMILES recovery

**Fig. 7.**
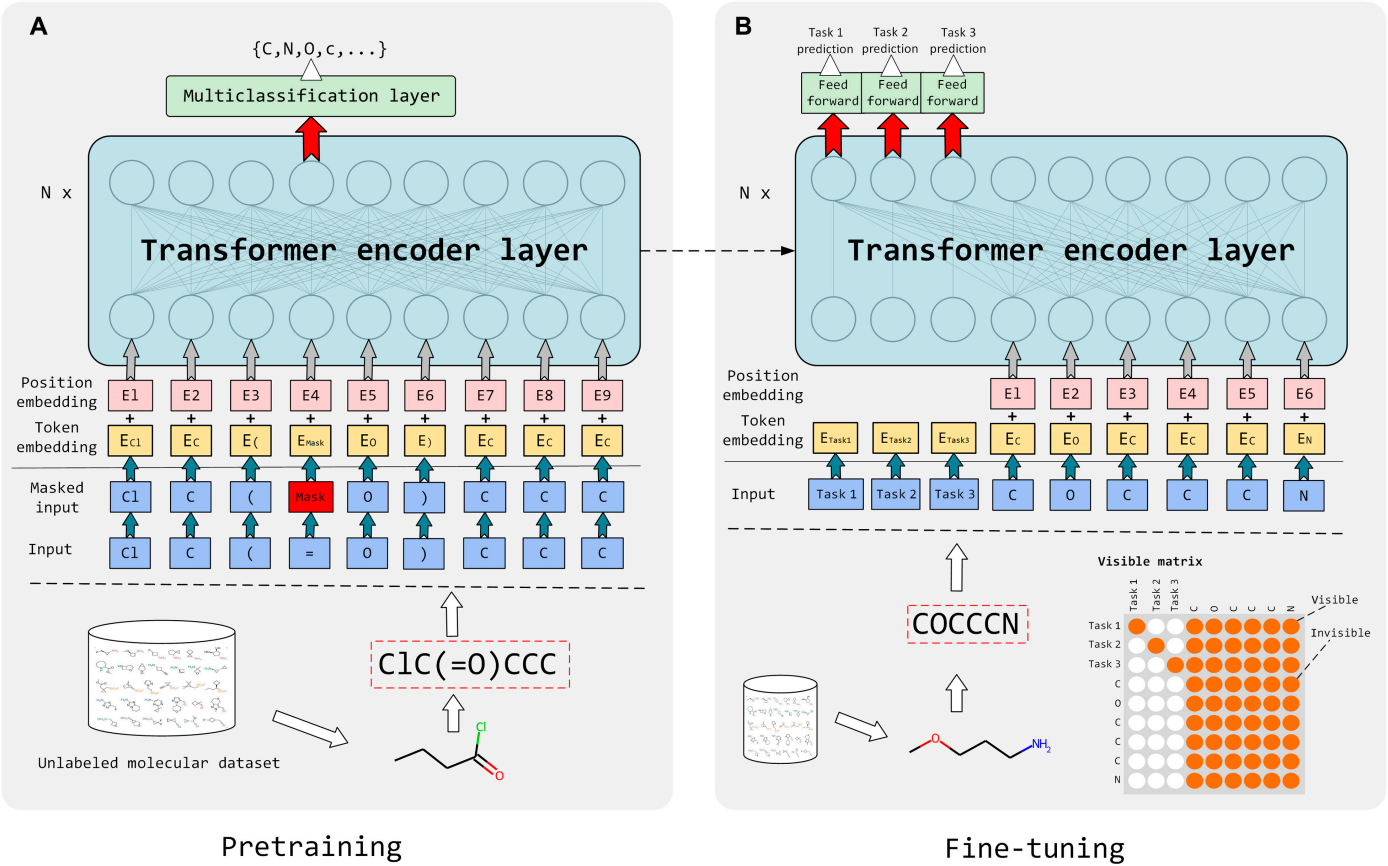
The schematic diagram of the (A) pretraining and (B) fine-tuning processes of MTL-BERT. Except for the output layer, the same architecture is used for pretraining and fine-tuning. In the pretraining stage, the masked token prediction task is used to mine the contextual information in SMILES strings. In the fine-tuning phase, the task tokens are used to extract information from the SMILES tokens for the related prediction tasks and the visible matrix is used to modulate the attention matrix in the Transformer layer to prevent direct information exchange in different tasks.

The schematic diagram of the pretraining stage is shown in Fig. 7A. The original BERT method uses a combination of 2 tasks (the masked language model task and the sequential sentences classification task) to pretrain the model. The masked language model task is to use the contextual words surrounding a mask token to predict what the masked word should be. The next sentence prediction task is to determine if 2 sentences are consecutive. However, unlike natural language, there is no continuous relationship between different SMILES strings. Roberta [46] proved that a good language model can be trained without using sequence relationships. Therefore, we just used the masked SMILES recovery task to pretrain our model.

Our pretraining strategy follows that of BERT to mask the input SMILES. Firstly, 15% of tokens in SMILES will be randomly selected, and at least one token will be selected for the molecules with only a few tokens. For each selected token, it has an 80% chance to be replaced by special masking tokens, a 10% chance to be randomly replaced by other tokens in the dictionary, and a 10% chance to be unchanged. The original SMILES is used as the ground truth to train the model, and the loss is only calculated at the masked tokens.

### Fine-tuning for multiple molecular property predictions

The schematic diagram of the fine-tuning stage is shown in Fig. 7B. After pretraining on a large number of unlabeled SMILES strings, the pretrained model has a nontrivial initialization. During the fine-tuning stage, we padded several task tokens based on the number of tasks before each input SMILES string. The model output at each of these tokens is paired with a task-related trainable feed-forward neural network and then used for different molecular property prediction tasks. Specifically, in the fine-tuning stage, we only added positional encoding to SMILES tokens to keep in accordance with the pretraining stage. Further, we also added an attention mask as shown in Fig. 7 to prevent task exchange information directly. Preventing the direct information exchange between task tokens allows the tasks to learn directly from SMILES tokens without causing interference between different tasks, especially when the number of tasks is quite large. Moreover, the inconsistency between pretraining and fine-tuning could be largely reduced, since the SMILES token cannot see the task token in the pretraining phase. Furthermore, it can also give the model better interpretability.

### Input representations

The SMILES strings consist of characters of variable length, which need to be processed into some tokens before they can be sent to the MTL-BERT model. We tokenized the SMILES strings using the following regular expression:

$$\begin{array}{c} “(\mathrm{Si}|\mathrm{Mg}|\mathrm{Ca}|\mathrm{Fe}|\mathrm{As}|\mathrm{Al}|\mathrm{Cl}|\mathrm{Br}|[\#\%\backslash )\backslash \\ \;(\backslash +\backslash -0123456789≔@\text{CBFIHONPS}\backslash [\backslash ]\text{icosn}]|/|\backslash \backslash )” \end{array}$$

The possible matched tokens, such as [Si], [Br], and [1], are added to the dictionary, and they basically cover the commonly used characters in the SMILES strings. To accommodate as many tasks as possible in the fine-tuning stage, 1,000 task-related tokens denoted as [T0], [T1],…, [T1,000] are further added to the dictionary. Besides, the [MASK] token is needed for representing the masked tokens in the pretraining stage and the [PAD] token allows SMILES token inputs of different lengths to be padded to form a batch.

### Model training and evaluation

In the pretraining stage, the MTL-BERT model was trained via the standard batch gradient descent algorithm with an Adam optimizer [47]. According to our experience, the learning rate was set to 1e−4, and the batch size was set to 512. The model was pretrained for 50 epochs, after which further training shows very trivial performance improvement. To evaluate the pretraining performance, the pretraining masking strategy was also used to mask the molecules from the test set, and then the recovery rate was calculated as the evaluation metric.

In the fine-tuning stage, for each output of the Transformer encoder layer corresponding to a task token, a 2-layer fully connected neural network was added. The dropout strategy was adopted to prevent overfitting [48]. The Adam optimizer was used as the fine-tuning optimizer. After hyperparameter tuning, the learning rate was set to 5e−5, the batch size was set to 64, and the dropout rate was set to 0.1. We used the cross-entropy loss function for classification tasks and the mean squares loss function for regression tasks. For the multitask learning losses, we did not optimize the weights between each task's losses, but rather simply added them up.

The regression models were evaluated by the square determination coefficient (*R*^2^) and root mean square error (RMSE). The classification models were evaluated by the area under the receiver operating characteristic (ROC-AUC) and accuracy. We used early stopping with patience 20 to prevent overfitting by monitoring the model loss for the validation dataset and set a maximum training epoch to 200. To reduce random errors, the entire experiment was trained 10 times with random data splitting, and the mean value and the standard deviation were reported as the final performance. To note, for the datasets containing more than one task, we just reported their average performance for simplicity.

## Data Availability

The original ChEMBL data can be accessed at https://www.ebi.ac.uk/chembl/. The MolecularNet data can be accessed at https://moleculenet.org/. The ADMETLab data can be accessed at https://admetmesh.scbdd.com/. Codes for MTL-BERT can be accessed at https://github.com/zhang-xuan1314/MTL-BERT.

## Supplementary Materials

Supplementary Materials Table S1. Detailed information of the 60 molecular property datasets used in this study. Table S2. Performance comparison (ROC-AUC and *R*^2^) of MTL-BERT, STL-BERT, and Cano-BERT (both are shown in percentage). Table S3. Performance comparison (accuracy and RMSE) of MTL-BERT, STL-BERT, and Cano-BERT (the classificaion task results are shown in percentage). Table S4. The performance comparison (ROC-AUC and *R*^2^) of the proposed model and state-of-the-art models (both are shown in percentage). Table S5. The performance comparison (accuracy and RMSE) of the proposed model and state-of-the-art models (the classificaion task results are shown in percentage).

## References

[1] Surabhi S, Singh B. Computer aided drug design: An overview. J Drug Deliv Ther. 2018;8(5):504–509.

[2] Simões RS, Maltarollo VG, Oliveira PR, Honorio KM. Transfer and multi-task learning in QSAR modeling: Advances and challenges. Front Pharmacol. 2018;9:74. 29467659 10.3389/fphar.2018.00074 PMC5807924

[3] Khanna I. Drug discovery in pharmaceutical industry: Productivity challenges and trends. Drug Discov Today. 2012;17(19–20):1088–1102. 22627006 10.1016/j.drudis.2012.05.007

[4] Vamathevan J, Clark D, Czodrowski P, Dunham I, Ferran E, Lee G, Li B, Madabhushi A, Shah P, Spitzer M, et al. Applications of machine learning in drug discovery and development. Nat Rev Drug Discov. 2019;18(6):463–477. 30976107 10.1038/s41573-019-0024-5 PMC6552674

[5] Song CM, Lim SJ, Tong JC. Recent advances in computer-aided drug design. Brief Bioinform. 2009;10(5):579–591. 19433475 10.1093/bib/bbp023

[6] David L, Thakkar A, Mercado R, Engkvist O. Molecular representations in AI-driven drug discovery: A review and practical guide. J Cheminform. 2020;12(1):56. 33431035 10.1186/s13321-020-00460-5 PMC7495975

[7] Shen J, Nicolaou CA. Molecular property prediction: Recent trends in the era of artificial intelligence. Drug Discov Today Technol. 2019;32–33:29–36. 10.1016/j.ddtec.2020.05.001 33386091

[8] Rogers D, Hahn M. Extended-connectivity fingerprints. J Chem Inf Model. 2010;50(5):742–754. 20426451 10.1021/ci100050t

[9] Sheridan RP, Wang WM, Liaw A, Ma J, Gifford EM. Extreme gradient boosting as a method for quantitative structure–activity relationships. J Chem Inf Model. 2016;56(12):2353–2360. 27958738 10.1021/acs.jcim.6b00591

[10] Gertrudes JC, Maltarollo VG, Silva RA, Oliveira PR, Honório KM, da Silva AB. Machine learning techniques and drug design. Curr Med Chem. 2012;19(25):4289–4297. 22830342 10.2174/092986712802884259

[11] Wieder O, Kohlbacher S, Kuenemann M, Garon A, Ducrot P, Seidel T, Langer T. A compact review of molecular property prediction with graph neural networks. Drug Discov Today Technol. 2020;37:1–12. 34895648 10.1016/j.ddtec.2020.11.009

[12] Wang J, Hsieh C-Y, Wang M, Wang X, Wu Z, Jiang D, Liao B, Zhang X, Yang B, He Q, et al. Multi-constraint molecular generation based on conditional transformer, knowledge distillation and reinforcement learning. Nat Mach Intell. 2021;3(10):914–922.

[13] Öztürk H, Özgür A, Ozkirimli E. DeepDTA: Deep drug–target binding affinity prediction. Bioinformatics. 2018;34(17):i821–i829. 30423097 10.1093/bioinformatics/bty593 PMC6129291

[14] Jumper J, Evans R, Pritzel A, Green T, Figurnov M, Ronneberger O, Tunyasuvunakool K, Bates R, Žídek A, Potapenko A, et al. Highly accurate protein structure prediction with AlphaFold. Nature. 2021;596(7873):583–589. 34265844 10.1038/s41586-021-03819-2 PMC8371605

[15] Karpov P, Godin G, Tetko IV. Transformer-CNN: Swiss knife for QSAR modeling and interpretation. J Cheminform. 2020;12(1):17. 33431004 10.1186/s13321-020-00423-w PMC7079452

[16] Wu CK, Zhang XC, Yang ZJ, Lu AP, Hou TJ, Cao DS. Learning to SMILES: BAN-based strategies to improve latent representation learning from molecules. Brief Bioinform. 2021;22(6):Article bbab327. 34427296 10.1093/bib/bbab327

[17] Xiong Z, Wang D, Liu X, Zhong F, Wan X, Li X, Li Z, Luo X, Chen K, Jiang H, et al. Pushing the boundaries of molecular representation for drug discovery with the graph attention mechanism. J Med Chem. 2020;63(16):8749–8760. 31408336 10.1021/acs.jmedchem.9b00959

[18] Ghasemi F, Mehridehnavi A, Pérez-Garrido A, Pérez-Sánchez H. Neural network and deep-learning algorithms used in QSAR studies: Merits and drawbacks. Drug Discov Today. 2018;23(10):1784–1790. 29936244 10.1016/j.drudis.2018.06.016

[19] Weininger D, Weininger A, Weininger JL. SMILES. 2. Algorithm for generation of unique SMILES notation. J Chem Inf Comput Sci. 1989;29(2):97–101.

[20] Yu Y, Si X, Hu C, Zhang J. A review of recurrent neural networks: LSTM cells and network architectures. Neural Comput. 2019;31(7):1235–1270. 31113301 10.1162/neco_a_01199

[21] Hu S, Chen P, Gu P, Wang B. A deep learning-based chemical system for QSAR prediction. IEEE J Biomed Health Inform. 2020;24(10):3020–3028. 32142459 10.1109/JBHI.2020.2977009

[22] Hong J, Luo Y, Mou M, Fu J, Zhang Y, Xue W, Xie T, Tao L, Lou Y, Zhu F. Convolutional neural network-based annotation of bacterial type IV secretion system effectors with enhanced accuracy and reduced false discovery. Brief Bioinform. 2020;21(5):1825–1836. 31860715 10.1093/bib/bbz120

[23] Krizhevsky A, Sutskever I, Hinton GE. Imagenet classification with deep convolutional neural networks. Adv Neural Inf Process Syst. 2012;25:1097–1105.

[24] Walters WP, Barzilay R. Applications of deep learning in molecule generation and molecular property prediction. Acc Chem Res. 2021;54(2):263–270. 33370107 10.1021/acs.accounts.0c00699

[25] Rong Y, Bian Y, Xu T, Xie W, Ying W, Huang W, Huang J. Self-supervised graph transformer on large-scale molecular data. arXiv. 2020. https://arxiv.org/abs/2007.02835.

[26] Zhuang F, Qi Z, Duan K, Xi D, Zhu Y, Zhu H, Xiong H, He Q. A comprehensive survey on transfer learning. Proc IEEE. 2020;109:43–76.

[27] Sosnin S, Vashurina M, Withnall M, Karpov P, Fedorov M, Tetko IV. A survey of multi-task learning methods in chemoinformatics. Mol Inform. 2019;38(4):Article 1800108. 30499195 10.1002/minf.201800108 PMC6587441

[28] Yu L, Su Y, Liu Y, Zeng X. Review of unsupervised pretraining strategies for molecules representation. Brief Funct Genomics. 2021;20(5):323–332. 34342611 10.1093/bfgp/elab036

[29] Zhang XC, Wu CK, Yang ZJ, Wu ZX, Yi JC, Hsieh CY, Hou TJ, Cao DS. MG-BERT: Leveraging unsupervised atomic representation learning for molecular property prediction. Brief Bioinform. 2021;22(6):Article bbab152. 33951729 10.1093/bib/bbab152

[30] Chen T, Kornblith S, Norouzi M, Hinton G. A simple framework for contrastive learning of visual representations. In: Blei D, editor. *Proceedings of the 37th international conference on machine learning*. PMLR: Virtual Event; 2020. Vol. 119, p. 1597–1607.

[31] Liu X, Zhang F, Hou Z, Wang Z, Mian L, Zhang J, Tang J. Self-supervised learning: Generative or contrastive. arXiv. 2020. https://arxiv.org/abs/2006.08218.

[32] Devlin J, Chang M-W, Lee K, Toutanova K. BERT: Pre-training of deep bidirectional transformers for language understanding. In: Burstein J, Doran C, Solorio T., editors. *Proceedings of the 2019 conference of the North American chapter of the Association for Computational Linguistics: Human language technologies*. Minneapolis (MN): Association for Computational Linguistics; 2018. Vol. 1. p. 4171–4186.

[33] Winter R, Montanari F, Noé F, Clevert DA. Learning continuous and data-driven molecular descriptors by translating equivalent chemical representations. Chem Sci. 2018;10(6):1692–1701. 30842833 10.1039/c8sc04175j PMC6368215

[34] Wang S, Guo Y, Wang J, Sun H, Huang J. SMILES-BERT: Large scale unsupervised pre-training for molecular property prediction. In: Xinghua MS, Michael B, Jian M, Pierangelo V, editors. *Proceedings of the 10th ACM International Conference on Bioinformatics, Computational Biology and Health Informatics; 2019 Sep 7–10; Niagara Falls (NY), USA,* p. 429–436.

[35] Honda S, Shi S, Ueda HR. Smiles transformer: Pre-trained molecular fingerprint for low data drug discovery. arXiv. 2019. https://arxiv.org/abs/1911.04738.

[36] Veličković P, Cucurull G, Casanova A, Romero A, Liò P, Bengio Y. Graph attention networks. Stat. 2018;1050:4.

[37] Kipf TN, Welling M. Semi-supervised classification with graph convolutional networks. arXiv. 2016. http://arxiv.org/abs/1609.02907.

[38] Van der Maaten L, Hinton G. Visualizing data using t-SNE. J Mach Learn Res. 2008;9:2579–2605.

[39] Plošnik A, Vračko M, Dolenc MS. Mutagenic and carcinogenic structural alerts and their mechanisms of action. Arh Hig Rada Toksikol. 2016;67(3):169–182. 27749264 10.1515/aiht-2016-67-2801

[40] Gaulton A, Bellis LJ, Bento AP, Chambers J, Davies M, Hersey A, Light Y, McGlinchey S, Michalovich D, Al-Lazikani B, et al. ChEMBL: A large-scale bioactivity database for drug discovery. Nucleic Acids Res. 2012;40(D1):D1100–D1107. 21948594 10.1093/nar/gkr777 PMC3245175

[41] Xiong G, Wu Z, Yi J, Fu L, Yang Z, Hsieh C, Yin M, Zeng X, Wu C, Lu A, et al. ADMETlab 2.0: An integrated online platform for accurate and comprehensive predictions of ADMET properties. Nucleic Acids Res. 2021;49(W1):W5–W14. 33893803 10.1093/nar/gkab255 PMC8262709

[42] Wu Z, Ramsundar B, Feinberg EN, Gomes J, Geniesse C, Pappu AS, Leswing K, Pande V. MoleculeNet: A benchmark for molecular machine learning. Chem Sci. 2017;9(2):513–530. 29629118 10.1039/c7sc02664a PMC5868307

[43] Hendrycks D, Gimpel K. Gaussian error linear units (GELUs). arxiv. 2016. https://arxiv.org/abs/1606.08415.

[44] Ba JL, Kiros JR, Hinton GE. Layer normalization. arXiv. 2016. https://arxiv.org/abs/1607.06450v1.

[45] Vaswani A, Shazeer N, Parmar N, Uszkoreit J, Jones L, Gomez AN, Kaiser Ł, Polosukhin I. Attention is all you need. In: Guyon UVLI, Bengio S, Wallach HM, Fergus Rob, Vishwanathan SVN, Garnett R, editors. *Advances in neural information processing systems 30: Annual conference on neural information processing systems 2017*; 2017 Dec 4–9; Long Beach, CA, p. 5998–6008.

[46] Liu Y, Ott M, Goyal N, Du J, Joshi M, Chen D, Levy O, Lewis M, Zettlemoyer L, Stoyanov V. Roberta: A robustly optimized bert pretraining approach. arXiv. 2019. https://arxiv.org/abs/1907.11692.

[47] Kingma DP, Ba J. Adam: A method for stochastic optimization. arXiv. 2015. https://arxiv.org/pdf/1412.6980.pdf.

[48] Srivastava N, Hinton G, Krizhevsky A, Sutskever I, Salakhutdinov R. Dropout: A simple way to prevent neural networks from overfitting. J Mach Learn Res. 2014;15(56):1929−1958.

