# Supplementary material for: Pushing the Boundaries of Molecular Property Prediction for Drug Discovery with Multitask Learning BERT Enhanced by SMILES Enumeration
Source: Research (Wash D C). 2022 Dec 15;2022:0004. doi: 10.34133/research.0004 (PMC11404312; doi:10.34133/research.0004)
Supplement: Supplementary Materials — Table S1. Detailed information of the 60 molecular property datasets used in this study. Table S2. Performance comparison (ROC-AUC and R2) of MTL-BERT, STL-BERT, and Cano-BERT (both are shown in percentage). Table S3. Performance comparison (accuracy and RMSE) of MTL-BERT, STL-BERT, and Cano-BERT (the classificaion task results are shown in percentage). Table S4. The performance comparison (ROC-AUC and R2) of the proposed model and state-of-the-art models (both are shown in percentage). Table S5. The performance comparison (accuracy and RMSE) of the proposed model and state-of-the-art models (the classificaion task results are shown in percentage). [file 0004.f1.docx]

Supplementary Materials

**Table-S1.** Detailed information of the 60 molecular property datasets used in this study.

| **Category** | **Dataset** | **Task Type** | **Number** | **Tasks** |
| --- | --- | --- | --- | --- |
| Absorption | Pgp-inhibitor | Classification | 2209 | 1 |
|  | Pgp-substrate | Classification | 1187 | 1 |
|  | HIA | Classification | 1160 | 1 |
|  | F_20%_ | Classification | 992 | 1 |
|  | F_30%_ | Classification | 992 | 1 |
|  | Caco-2 permeability | Regression | 2464 | 1 |
|  | MDCK permeability | Regression | 1140 | 1 |
| Distribution | BBBP | Classification | 1963 | 1 |
|  | PPB | Regression | 4712 | 1 |
|  | VD | Regression | 1086 | 1 |
|  | Fu | Regression | 2575 | 1 |
| Metabolism | CYP1A2 inhibitor | Classification | 12614 | 1 |
|  | CYP1A2 substrate | Classification | 366 | 1 |
|  | CYP2C19 inhibitor | Classification | 12611 | 1 |
|  | CYP2C19 substrate | Classification | 256 | 1 |
|  | CYP2C9 inhibitor | Classification | 12089 | 1 |
|  | CYP2C9 substrate | Classification | 811 | 1 |
|  | CYP2D6 inhibitor | Classification | 13073 | 1 |
|  | CYP2D6 substrate | Classification | 877 | 1 |
|  | CYP3A4 inhibitor | Classification | 12339 | 1 |
|  | CYP3A4 substrate | Classification | 979 | 1 |
| Excretion | T_1/2_ | Classification | 1219 | 1 |
|  | CL | Regression | 831 | 1 |
| Toxicity | ClinTox | Classification | 1464 | 2 |
|  | Sider | Classification | 1292 | 27 |
|  | hERG Blockers | Classification | 13845 | 1 |
|  | H-HT | Classification | 2304 | 1 |
|  | DILI | Classification | 467 | 1 |
|  | AMES Toxicity | Classification | 7575 | 1 |
|  | Rat Oral Acute Toxicity Toxicity | Classification | 7327 | 1 |
|  | FDAMDD | Classification | 1197 | 1 |
|  | Skin Sensitization | Classification | 405 | 1 |
|  | Carcinogencity | Classification | 1041 | 1 |
|  | Eye Corrosion | Classification | 2298 | 1 |
|  | Eye Irritation | Classification | 5219 | 1 |
|  | Respiratory Toxicity | Classification | 1388 | 1 |
|  | NR-AR | Classification | 7312 | 1 |
|  | NR-AR-LBD | Classification | 6862 | 1 |
|  | NR-AhR | Classification | 6603 | 1 |
|  | NR-Aromatase | Classification | 5887 | 1 |
|  | NR-ER | Classification | 6166 | 1 |
|  | NR-ER-LBD | Classification | 7052 | 1 |
|  | NR-PPAR-gamma | Classification | 6586 | 1 |
|  | SR-ARE | Classification | 5652 | 1 |
|  | SR-ATAD5 | Classification | 7170 | 1 |
|  | SR-HSE | Classification | 6319 | 1 |
|  | SR-MMP | Classification | 5913 | 1 |
|  | SR-p53 | Classification | 6915 | 1 |
|  | Bioconcentration factor | Regression | 676 | 1 |
|  | IGC_50_ | Regression | 1787 | 1 |
|  | LC_50_FM | Regression | 816 | 1 |
|  | LC_50_DM | Regression | 347 | 1 |
| Biophysics | HIV | Classification | 40890 | 1 |
|  | BACE | Classification | 1510 | 1 |
| Physicochemical property | ESOL | Regression | 1110 | 1 |
|  | FreeSolv | Regression | 639 | 1 |
|  | Lipophilicity | Regression | 4182 | 1 |
|  | LogS | Regression | 4797 | 1 |
|  | LogD_7.4_ | Regression | 10370 | 1 |
|  | LogP | Regression | 12682 | 1 |

**Table S2.** Performance comparison (ROC-AUC, R2) of MTL-BERT, STL-BERT and Cano-BERT (Both are showed in percentage).

| **Datasets** | **MTL-BERT** | **STL-BERT** | **Cano-BERT** |
| --- | --- | --- | --- |
| **Classification tasks** | | | |
| Pgp-inh | **93.7±1.5** | 92.1±2.2 | 92.1±0.7 |
| Pgp-sub | **92.1±1.8** | 92.0±2.5 | 89.3±3.2 |
| HIA | **89.9±4.5** | 87.3±7.5 | 77.1±4.1 |
| F20% | **83.2±4.6** | 73.5±5.6 | 69.7±3.5 |
| F30% | **81.4±5.2** | 77.4±4.0 | 75.7±5.1 |
| BBBP | **95.9±1.0** | 93.1±2.1 | 92.1±2.3 |
| CYP1A2-inh | **94.5±0.4** | 93.9±0.6 | 93.2±0.6 |
| CYP1A2-sub | 80.8±6.6 | **84.1±6.3** | 77.1±4.5 |
| CYP2C19-inh | **91.7±0.8** | 90.7±0.6 | 89.5±0.6 |
| CYP2C19-sub | **78.0±6.9** | 75.0±6.9 | 77.1±10.1 |
| CYP2C9-inh | **91.9±0.8** | 90.2±1.0 | 89.1±0.6 |
| CYP2C9-sub | **77.4±5.0** | 73.9±4.4 | 70.8±7.3 |
| CYP2D6-inh | **90.3±0.8** | 89.5±1.3 | 86.9±1.7 |
| CYP2D6-sub | **82.0±5.2** | 81.9±2.7 | 79.8±4.4 |
| CYP3A4-inh | **92.0±0.9** | 91.1±0.8 | 90.1±0.6 |
| CYP3A4-sub | **82.1±4.1** | 80.2±5.6 | 78.1±4.6 |
| T1/2 | **76.6±6.6** | 75.2±4.6 | 73.8±4.5 |
| ClinTox | **94.8±4.4** | 87.4±7.5 | 84.6±6.5 |
| Sider | **66.1±4.0** | 63.6±2.6 | 62.5±6.7 |
| hERG Blockers | 94.0±1.0 | **94.5±0.6** | 93.1±0.4 |
| H-HT | **79.1±3.7** | 75.7±2.4 | 73.9±3.2 |
| DILI | 89.8±3.8 | **90.9±3.6** | 86.4±4.4 |
| AMES | 89.5±0.6 | **90.2±1.4** | 88.8±1.3 |
| Rat Oral Acute Toxicity | 86.0±1.4 | **86.8±0.8** | 85.7±1.4 |
| FDAMDD | 83.0±2.8 | **83.6±3.7** | 83.3±3.4 |
| Skin Sensitization | 82.7±4.2 | **82.9±5.2** | 78.4±6.0 |
| Carcinogencity | **73.5±5.4** | 70.3±6.5 | 67.3±3.9 |
| Eye Corrosion | **99.4±0.3** | 99.2±0.5 | 99.1±0.7 |
| Eye Irritation | **97.9±0.5** | **97.9±1.0** | 97.5±1.1 |
| Respiratory Toxicity | **89.2±2.0** | 87.8±2.3 | 84.3±3.6 |
| NR-AR | **90.2±4.4** | 86.4±3.2 | 82.1±5.6 |
| NR-AR-LBD | **97.0±2.4** | 89.2±4.9 | 88.7±3.6 |
| NR-AhR | **95.0±1.7** | 91.9±0.9 | 91.8±1.3 |
| NR-Aromatase | **92.2±1.3** | 86.4±4.4 | 83.3±5.3 |
| NR-ER | **85.4±3.1** | 78.8±2.7 | 76.9±2.9 |
| NR-ER-LBD | **92.6±2.5** | 86.7±1.8 | 85.0±3.4 |
| NR-PPAR-gamma | **94.6±1.6** | 82.7±4.4 | 80.1±5.0 |
| SR-ARE | **91.8±1.5** | 84.8±1.5 | 82.8±2.7 |
| SR-ATAD5 | **95.2±2.0** | 83.0±4.2 | 84.7±4.6 |
| SR-HSE | **89.8±3.8** | 82.2±2.9 | 80.7±4.5 |
| SR-MMP | **96.7±0.7** | 92.0±2.2 | 91.2±1.8 |
| SR-p53 | **94.6±1.7** | 87.2±2.0 | 83.2±1.5 |
| HIV | **83.4±2.0** | 83.1±1.7 | 83.3±2.8 |
| BACE | 87.7±3.0 | **88.3±2.0** | 85.7±2.1 |
| **Regression tasks** | | | |
| Caco-2 | 80.0±3.1 | **81.2±3.2** | 75.0±3.9 |
| MDCK | **70.8±6.5** | 69.7±4.3 | 59.3±10.7 |
| PPB | **83.6±2.0** | 78.7±2.7 | 64.4±3.2 |
| VD | **73.5±8.1** | 64.1±17.5 | 56.2±6.9 |
| Fu | **79.5±2.6** | 66.5±3.9 | 56.1±5.2 |
| CL | **62.5±8.4** | 57.2±14.0 | 53.6±10.4 |
| Bioconcentration Factor | **82.7±4.0** | 79.0±5.4 | 74.4±6.1 |
| IGC50 | **85.7±2.0** | 85.2±2.1 | 75.1±1.8 |
| LC50FM | **70.0±7.9** | 68.6±9.6 | 60.6±9.2 |
| LC50DM | **74.7±5.6** | 64.2±14.4 | 57.4±9.2 |
| ESOL | **94.4±1.1** | 91.5±2.4 | 87.8±3.1 |
| FreeSolv | 92.8±5.2 | **93.1±2.7** | 88.8±3.6 |
| Lipophilicity | **84.7±3.0** | 78.9±1.7 | 67.0±3.7 |
| LogS | **88.1±1.6** | 86.1±1.8 | 83.8±1.5 |
| LogD | **90.7±0.6** | 88.5±0.8 | 77.6±2.2 |
| LogP | **95.9±0.2** | 95.0±0.5 | 91.7±0.5 |

**Table S3.** Performance comparison (Accuracy, RMSE) of MTL-BERT, STL-BERT and Cano-BERT (The classificaion task results are showed in percentage).

| **Datasets** | **MTL-BERT** | **STL-BERT** | **Cano-BERT** |
| --- | --- | --- | --- |
| **Classification tasks** | | | |
| Pgp-inh | **86.1±2.0** | 85.1±2.6 | 83.3±2.5 |
| Pgp-sub | **84.0±2.4** | 83.9±2.7 | 82.5±3.6 |
| HIA | 89.8±3.1 | **90.0±2.3** | 87.3±1.2 |
| F20% | **80.5±5.3** | 75.2±4.8 | 74.7±4.2 |
| F30% | **74.5±5.47** | 71.5±4.0 | 72.0±4.0 |
| BBBP | **93.0±1.6** | 89.6±1.9 | 89.2±1.6 |
| CYP1A2-inh | **87.0±1.1** | 86.4±1.1 | 85.1±0.5 |
| CYP1A2-sub | **71.4±6.2** | 71.1±8.0 | 65.9±6.5 |
| CYP2C19-inh | **83.9±1.0** | 83.8±0.7 | 82.0±0.8 |
| CYP2C19-sub | **70.4±5.8** | 68.1±11.0 | 64.6±12.1 |
| CYP2C9-inh | **84.7±1.1** | 83.0±1.1 | 82.1±1.1 |
| CYP2C9-sub | **71.0±4.6** | 66.7±5.6 | 66.2±7.1 |
| CYP2D6-inh | **88.6±0.6** | **88.6±1.1** | 87.3±0.9 |
| CYP2D6-sub | 74.3±6.1 | **74.9±3.7** | 72.9±4.1 |
| CYP3A4-inh | **83.2±1.4** | 82.3±1.0 | 81.3±0.9 |
| CYP3A4-sub | **73.6±4.9** | 73.3±4.9 | 69.4±4.8 |
| T1/2 | **69.8±5.4** | 67.3±3.2 | 68.3±4.4 |
| ClinTox | **96.1±1.4** | 95.2±1.3 | 93.5±2.0 |
| Sider | **75.6±3.5** | 74.9±1.3 | 74.9±3.2 |
| hERG Blockers | 88.2±1.2 | **88.7±1.1** | 87.0±1.0 |
| H-HT | **70.4±3.8** | 67.5±2.1 | 66.9±3.3 |
| DILI | 82.3±5.8 | **83.0±4.7** | 80.9±5.9 |
| AMES | 83.0±1.6 | **83.4±1.5** | 82.4±1.7 |
| Rat Oral Acute Toxicity | 78.3±1.1 | **79.0±1.5** | 78.6±1.7 |
| FDAMDD | 74.8±4.5 | **77.2±3.6** | 76.9±3.1 |
| Skin Sensitization | **77.9±3.9** | 76.3±7.1 | 72.5±5.3 |
| Carcinogencity | **66.9±4.6** | 65.3±5.0 | 60.2±4.2 |
| Eye Corrosion | 96.2±1.1 | **96.9±1.1** | 96.6±1.0 |
| Eye Irritation | 94.2±0.6 | **94.7±1.5** | 93.8±1.3 |
| Respiratory Toxicity | **80.1±3.0** | **80.1±3.4** | 74.9±4.0 |
| NR-AR | **98.2±0.6** | 98.1±0.5 | 98.0±0.4 |
| NR-AR-LBD | **98.2±0.5** | 97.8±0.7 | 97.9±0.4 |
| NR-AhR | **94.2±0.6** | 92.1±0.7 | 91.7±0.6 |
| NR-Aromatase | **96.4±0.7** | 96.3±0.6 | 95.8±0.9 |
| NR-ER | **93.0±0.7** | 91.0±1.4 | 90.8±1.0 |
| NR-ER-LBD | **97.0±0.5** | 96.9±0.6 | 96.2±0.8 |
| NR-PPAR-gamma | **97.6±0.5** | 97.3±0.6 | 97.2±0.6 |
| SR-ARE | **90.5±1.2** | 86.6±1.7 | 85.9±1.0 |
| SR-ATAD5 | **97.6±0.6** | 96.5±0.3 | 96.5±0.5 |
| SR-HSE | **96.2±0.7** | 94.7±0.8 | 95.0±0.7 |
| SR-MMP | **94.0±1.2** | 90.6±2.0 | 90.6±1.2 |
| SR-p53 | **95.4±1.0** | 93.1±0.8 | 93.5±1.4 |
| HIV | **97.2±0.2** | **97.2±0.2** | 97.1±0.2 |
| BACE | 80.5±5.0 | **80.9±2.5** | 79.5±2.5 |
| Regression tasks | | | |
| Caco-2 | 0.311±0.075 | **0.285±0.015** | 0.327±0.016 |
| MDCK | **0.334±0.107** | 0.348±0.037 | 0.367±0.051 |
| PPB | **9.373±4.743** | 12.298±0.909 | 17.042±0.900 |
| VD | **0.773±0.253** | 0.903±0.264 | 0.945±0.125 |
| Fu | **0.356±0.057** | 0.429±0.030 | 0.487±0.027 |
| CL | **2.523±1.080** | 3.273±0.609 | 3.438±0.445 |
| Bioconcentration Factor | **0.553±0.091** | 0.629±0.054 | 0.689±0.069 |
| IGC50 | **0.377±0.033** | 0.397±0.031 | 0.540±0.017 |
| LC50FM | **0.745±0.118** | 0.774±0.072 | 0.934±0.088 |
| LC50DM | **0.786±0.210** | 0.873±0.174 | 1.225±0.224 |
| ESOL | **0.437±0.106** | 0.590±0.072 | 0.731±0.077 |
| FreeSolv | 0.950±0.539 | **0.949±0.228** | 1.316±0.182 |
| Lipophilicity | **0.446±0.061** | 0.544±0.025 | 0.701±0.027 |
| LogS | **0.682±0.177** | 0.816±0.046 | 0.881±0.030 |
| LogD | **0.394±0.048** | 0.472±0.015 | 0.680±0.030 |
| LogP | **0.338±0.070** | 0.406±0.024 | 0.531±0.013 |

**Table S4.** The performance comparison (ROC-AUC, R2) of the proposed model and state-of-the-art models (Both are showed in percentage).

|  | **ECFP-XGBoost** | **GAT** | **GCN** | **AttentiveFP** | **CDDD** | **MTL-BERT** |
| --- | --- | --- | --- | --- | --- | --- |
| Classification tasks | | | | | | |
| Pgp-inh | 89.8±1.5 | 91.1±2.0 | 92.6±1.5 | 91.8±2.6 | 91.9±2.0 | **93.7±1.5** |
| Pgp-sub | 86.8±1.8 | 88.3±2.2 | 89.3±2.0 | 89.9±2.6 | 90.8±2.7 | **92.1±1.8** |
| HIA | 78.4±8.4 | 83.6±6.6 | 84.1±7.5 | 85.1±5.4 | 85.3±5.8 | **89.9±4.5** |
| F20% | 71.1±6.9 | 72.4±6.1 | 71.7±6.1 | 69.8±4.6 | 75.1±7.6 | **83.2±4.6** |
| F30% | 77.2±5.5 | 67.8±3.6 | 68.2±5.8 | 69.8±5.4 | 71.2±6.5 | **81.4±5.2** |
| BBBP | 91.2±2.8 | 91.7±3.4 | 89.7±1.8 | 90.3±2.4 | 90.2±2.4 | **95.9±1.0** |
| CYP1A2-inh | 90.9±0.4 | 92.0±0.7 | 92.6±0.8 | 93.3±0.7 | 92.6±0.4 | **94.5±0.4** |
| CYP1A2-sub | 73.9±9.4 | 77.2±10.0 | 75.8±7.8 | 76.1±9.5 | 74.7±8.1 | **80.8±6.6** |
| CYP2C19-inh | 87.6±0.8 | 87.6±0.4 | 89.0±0.5 | 89.4±0.6 | 88.6±0.8 | **91.7±0.8** |
| CYP2C19-sub | 71.4±12.4 | 65.1±9.2 | 73.7±7.0 | 72.9±10.0 | **79.0±10.5** | 78.0±6.9 |
| CYP2C9-inh | 87.4±1.2 | 88.3±1.4 | 89.3±1.2 | 89.7±1.1 | 89.0±0.6 | **91.9±0.8** |
| CYP2C9-sub | 74.8±4.4 | 72.4±5.0 | 75.8±7.0 | 69.5±6.5 | 69.8±5.8 | **77.4±5.0** |
| CYP2D6-inh | 85.8±1.6 | 87.4±1.1 | 88.5±1.3 | 89.1±1.1 | 87.1±0.9 | **90.3±0.8** |
| CYP2D6-sub | 76.0±5.4 | 72.1±5.9 | 80.6±3.3 | 74.9±2.9 | 77.3±7.1 | **82.0±5.2** |
| CYP3A4-inh | 86.9±1.0 | 89.7±0.8 | 90.2±0.9 | 91.3±0.8 | 89.2±1.1 | **92.0±0.9** |
| CYP3A4-sub | 75.4±5.3 | 79.6±3.6 | 80.0±5.4 | 79.8±4.4 | 79.0±5.2 | **82.1±4.1** |
| T1/2 | 71.3±6.9 | 72.0±5.9 | 72.0±4.7 | 72.5±4.5 | 74.5±3.2 | **76.6±6.6** |
| ClinTox | 85.5±7.5 | 89.4±5.4 | 90.1±7.9 | 91.7±5.9 | 93.2±5.4 | **94.8±4.4** |
| Sider | 63.2±2.1 | 61.5±1.9 | 60.9±1.1 | 60.7±2.9 | 58.2±2.8 | **66.1±4.0** |
| hERG Blockers | 92.0±0.8 | 92.4±0.8 | 92.6±0.7 | 93.4±0.5 | 92.6±0.8 | **94.0±1.0** |
| H-HT | 77.3±3.4 | 75.6±3.0 | 75.8±2.4 | 74.7±3.0 | 75.7±2.1 | **79.1±3.7** |
| DILI | 85.2±6.2 | 84.6±6.3 | 86.3±4.6 | 86.2±5.1 | 87.9±4.0 | **89.8±3.8** |
| AMES | 87.7±1.1 | 88.5±1.2 | 88.9±0.8 | 88.8±1.5 | 88.3±1.0 | **89.5±0.6** |
| Rat Oral Acute Toxicity | 83.4±1.5 | 83.2±2.1 | 85.3±1.8 | 84.7±1.8 | 84.4±0.7 | **86.0±1.4** |
| FDAMDD | 82.8±4.5 | 80.4±3.9 | 80.6±3.7 | 79.9±2.7 | 81.6±4.1 | **83.0±2.8** |
| Skin Sensitization | 63.0±9.3 | 72.3±11.3 | 71.2±5.7 | 69.4±9.8 | 75.8±5.5 | **82.7±4.2** |
| Carcinogencity | 73.1±2.7 | 66.1±3.8 | 69.6±4.4 | 69.4±3.2 | 70.1±6.4 | **73.5±5.4** |
| Eye Corrosion | 98.2±0.7 | 98.5±1.1 | 99.1±0.6 | 99.0±0.5 | 99.1±0.5 | **99.4±0.3** |
| Eye Irritation | 95.8±1.6 | 97.2±0.8 | 97.0±0.5 | 97.3±0.9 | 97.4±1.1 | **97.9±0.5** |
| Respiratory Toxicity | 82.0±4.3 | 82.9±4.6 | 85.7±2.5 | 83.6±3.7 | 85.3±2.2 | **89.2±2.0** |
| NR-AR | 79.0±5.4 | 82.8±8.7 | 84.5±8.5 | 85.1±6.9 | 83.4±3.6 | **90.2±4.4** |
| NR-AR-LBD | 86.0±4.7 | 89.4±4.3 | 90.5±3.4 | 89.0±5.0 | 89.6±4.3 | **97.0±2.4** |
| NR-AhR | 86.5±2.8 | 89.2±1.7 | 89.0±1.6 | 90.5±1.7 | 90.2±2.4 | **95.0±1.7** |
| NR-Aromatase | 75.9±4.2 | 82.2±4.5 | 82.4±3.4 | 84.2±4.0 | 87.5±2.9 | **92.2±1.3** |
| NR-ER | 73.9±4.2 | 77.2±2.5 | 76.8±2.5 | 76.9±3.1 | 79.7±3.1 | **85.4±3.1** |
| NR-ER-LBD | 82.7±3.9 | 82.0±5.2 | 83.6±3.7 | 84.8±5.6 | 88.4±3.4 | **92.6±2.5** |
| NR-PPAR-gamma | 74.7±3.6 | 83.2±5.7 | 84.4±5.9 | 83.0±5.9 | 85.5±6.7 | **94.6±1.6** |
| SR-ARE | 73.7±3.0 | 83.1±2.3 | 82.7±2.7 | 83.5±1.7 | 83.0±2.1 | **91.8±1.5** |
| SR-ATAD5 | 76.7±5.2 | 81.5±6.1 | 79.8±3.9 | 80.8±3.1 | 84.6±4.2 | **95.2±2.0** |
| SR-HSE | 70.2±4.8 | 78.4±4.7 | 81.7±4.2 | 81.0±4.4 | 78.2±6.6 | **89.8±3.8** |
| SR-MMP | 84.7±2.7 | 90.2±1.1 | 91.3±1.3 | 91.3±1.2 | 90.9±1.9 | **96.7±0.7** |
| SR-p53 | 73.3±2.2 | 84.3±3.3 | 84.3±4.1 | 82.2±2.9 | 84.1±2.1 | **94.6±1.7** |
| HIV | 78.6±2.2 | 82.5±1.6 | 82.6±2.1 | 79.9±2.3 | 79.8±2.3 | **83.4±2.0** |
| BACE | 88.3±2.0 | 87.7±2.8 | **88.8±1.8** | 84.6±3.3 | 86.8±1.5 | 87.7±3.0 |
| Regression tasks | | | | | | |
| Caco-2 | 66.8±4.4 | 65.5±9.8 | 72.7±5.1 | 72.4±4.0 | 75.3±3.6 | **80.0±3.1** |
| MDCK | 59.2±9.7 | 62.0±8.6 | 53.1±15.8 | 48.6±14.0 | 65.2±8.0 | **70.8±6.5** |
| PPB | 60.9±3.5 | 69.4±3.6 | 71.1±2.2 | 69.4±3.8 | 69.2±3.0 | **83.6±2.0** |
| VD | 57.7±15.8 | 44.1±17.0 | 44.2±17.5 | 45.2±24.4 | 50.4±12.2 | **73.5±8.1** |
| Fu | 60.5±3.7 | 58.7±4.1 | 56.4±4.5 | 63.7±4.6 | 66.3±3.6 | **79.5±2.6** |
| CL | 58.7±17.2 | 39.3±22.5 | 53.9±14.1 | 38.3±13.3 | 38.9±24.1 | **62.5±8.4** |
| Bioconcentration Factor | 56.0±7.0 | 66.2±8.8 | 60.3±11.7 | 74.6±7.4 | 71.9±7.9 | **82.7±4.0** |
| IGC50 | 54.6±5.5 | 77.9±8.4 | 80.0±3.3 | 83.5±2.5 | 81.5±3.0 | **85.7±2.0** |
| LC50FM | 48.2±8.8 | 59.4±9.3 | 62.7±8.5 | 61.7±6.9 | 60.9±10.0 | **70.0±7.9** |
| LC50DM | 47.9±12.3 | 50.6±14.3 | 46.8±18.3 | 41.6±17.1 | 59.0±12.2 | **74.7±5.6** |
| ESOL | 63.6±5.6 | 84.5±5.7 | 87.4±5.6 | 90.8±3.3 | 91.6±1.3 | **94.4±1.1** |
| FreeSolv | 60.6±8.3 | 84.4±6.3 | 88.2±5.3 | 91.5±2.3 | 92.2±2.0 | **92.8±5.2** |
| Lipophilicity | 52.3±3.5 | 70.8±1.9 | 70.9±3.9 | 76.6±2.4 | 66.4±4.0 | **84.7±3.0** |
| LogS | 68.4±2.7 | 84.4±1.9 | 85.3±2.3 | 86.2±1.7 | 85.6±1.6 | **88.1±1.6** |
| LogD | 74.3±2.2 | 78.7±2.0 | 86.9±0.9 | 89.6±0.7 | 82.9±1.5 | **90.7±0.6** |
| LogP | 79.1±0.9 | 94.2±0.5 | 95.2±0.5 | 95.7±0.5 | 91.8±0.8 | **95.9±0.2** |

**Table S5.** The performance comparison (Accuracy, RMSE) of the proposed model and state-of-the-art models (The classificaion task results are showed in percentage).

|  | **ECFP-XGBoost** | **GAT** | **GCN** | **AttentiveFP** | **CDDD** | **MTL-BERT** |
| --- | --- | --- | --- | --- | --- | --- |
| Classification tasks | | | | | | |
| Pgp-inh | 82.6±2.1 | 79.6±7.9 | 84.4±1.8 | 84.1±3.3 | 84.8±2.1 | **86.1±2.0** |
| Pgp-sub | 78.0±2.5 | 74.8±3.3 | 81.5±2.7 | 83.4±3.8 | 82.8±3.7 | **84.0±2.4** |
| HIA | 77.8±4.5 | 71.8±5.9 | 85.5±3.9 | 77.0±8.3 | 85.3±4.1 | **89.8±3.2** |
| F20% | 71.3±5.1 | 66.5±4.9 | 69.8±4.7 | 70.6±5.8 | 73.3±5.2 | **80.5±5.3** |
| F30% | 73.7±4.4 | 62.3±6.1 | 61.1±7.9 | 65.5±4.5 | 67.2±5.4 | **74.5±5.5** |
| BBBP | 84.1±2.6 | 81.2±4.6 | 85.0±4.9 | 85.0±3.6 | 86.6±2.6 | **93.0±1.6** |
| CYP1A2-inh | 82.8±0.7 | 83.0±1.2 | 85.3±1.0 | 85.9±1.0 | 84.9±1.0 | **87.0±1.2** |
| CYP1A2-sub | 67.3±8.3 | 59.7±12.5 | 64.9±6.4 | 62.7±12.7 | 69.5±7.8 | **71.4±6.2** |
| CYP2C19-inh | 79.8±1.1 | 78.0±3.1 | 81.4±1.2 | 82.2±0.8 | 81.3±01.3 | **83.9±1.0** |
| CYP2C19-sub | 67.7±9.6 | 63.1±7.3 | 68.8±3.8 | 69.2±11.3 | **72.1±11.0** | 70.4±5.8 |
| CYP2C9-inh | 79.7±1.3 | 78.4±3.4 | 81.3±1.4 | 81.7±1.6 | 82.2±1.2 | **84.7±1.0** |
| CYP2C9-sub | 68.9±4.1 | 63.7±7.7 | 67.8±6.5 | 62.4±6.4 | 66.2±4.6 | **71.0±4.6** |
| CYP2D6-inh | 82.8±1.5 | 79.7±1.0 | 83.7±1.3 | 83.7±2.4 | 82.8±1.9 | **88.6±0.6** |
| CYP2D6-sub | 69.4±4.0 | 54.8±8.8 | 73.6±3.6 | 67.4±3.9 | 71.9±4.9 | **74.3±6.1** |
| CYP3A4-inh | 78.5±1.3 | 77.8±2.8 | 82.0±1.1 | 82.9±1.2 | 80.8±1.2 | **83.2±1.4** |
| CYP3A4-sub | 69.2±5.7 | 66.4±6.6 | 70.6±4.6 | 72.1±3.5 | 72.3±4.9 | **73.6±4.9** |
| T1/2 | 65.2±6.6 | 66.2±5.6 | 66.5±4.4 | 66.7±4.5 | 68.3±4.3 | **69.8±5.4** |
| ClinTox | 87.7±9.2 | 82.4±8.6 | 83.9±7.2 | 89.7±8.9 | 91.8±5.7 | **96.1±1.4** |
| Sider | 64.7±2.3 | 59.2±3.4 | 59.5±2.2 | 62.3±3.6 | 58.7±4.9 | **75.6±3.5** |
| hERG Blockers | 84.9±1.1 | 83.2±4.7 | 85.4±0.8 | 86.9±0.7 | 86.0±1.2 | **88.2±1.2** |
| H-HT | 69.1±3.7 | 68.3±2.6 | 69.5±2.4 | 66.2±2.9 | 68.8±2.2 | **70.4±3.8** |
| DILI | 76.4±6.0 | 73.0±10.1 | 75.1±9.7 | 80.9±5.4 | 80.4±3.1 | **82.3±5.8** |
| AMES | 79.7±1.4 | 79.7±2.2 | 81.7±1.0 | 81.9±1.4 | 81.5±1.4 | **83.0±1.6** |
| Rat Oral Acute Toxicity | 76.7±1.4 | 73.5±4.7 | 78.0±1.5 | 76.9±2.2 | 76.7±1.7 | **78.3±1.1** |
| FDAMDD | 74.6±4.4 | 67.4±8.2 | 72.7±3.7 | 72.9±3.5 | 73.3±4.3 | **74.8±4.5** |
| Skin Sensitization | 63.9±8.6 | 69.4±9.4 | 67.8±6.8 | 63.4±8.5 | 70.8±6.0 | **77.9±3.9** |
| Carcinogencity | 65.3±3.6 | 60.2±4.1 | 64.0±4.5 | 63.5±3.8 | 63.8±5.0 | **66.9±4.6** |
| Eye Corrosion | 93.6±2.2 | 94.0±3.3 | 96.4±1.2 | 95.2±1.5 | 95.6±1.8 | **96.2±1.1** |
| Eye Irritation | 92.4±1.6 | 92.8±2.0 | 93.8±1.3 | 93.2±1.5 | 93.9±2.0 | **94.2±0.6** |
| Respiratory Toxicity | 73.4±4.9 | 73.2±9.4 | 78.9±2.7 | 77.7±4.1 | 78.1±3.5 | **80.1±3.0** |
| NR-AR | 93.5±4.2 | 83.7±6.6 | 84.6±9.2 | 89.4±8.7 | 92.0±4.1 | **98.2±0.6** |
| NR-AR-LBD | 96.4±1.3 | 84.3±14.2 | 91.6±2.9 | 91.5±7.1 | 92.5±2.3 | **98.2±0.5** |
| NR-AhR | 85.0±2.5 | 78.1±8.3 | 82.1±4.1 | 83.0±4.1 | 86.0±3.4 | **94.2±0.6** |
| NR-Aromatase | 86.6±3.1 | 77.7±11.7 | 81.3±8.0 | 79.5±8.4 | 87.9±5.9 | **96.4±0.7** |
| NR-ER | 73.8±5.6 | 70.4±7.3 | 73.3±6.7 | 82.5±4.6 | 84.1±6.4 | **93.0±0.7** |
| NR-ER-LBD | 89.2±2.1 | 86.3±8.6 | 84.7±5.3 | 83.9±4.5 | 87.6±7.6 | **97.0±0.5** |
| NR-PPAR-gamma | 88.0±4.3 | 83.4±6.2 | 85.7±7.1 | 76.6±5.7 | 89.3±5.5 | **97.6±0.5** |
| SR-ARE | 76.9±3.2 | 79.6±2.2 | 81.9±3.0 | 77.1±3.5 | 81.3±4.1 | **90.5±1.2** |
| SR-ATAD5 | 90.5±3.5 | 86.3±5.3 | 80.5±6.7 | 80.9±7.9 | 89.8±7.0 | **97.6±0.6** |
| SR-HSE | 85.5±3.4 | 67.7±5.3 | 82.6±6.0 | 80.6±5.9 | 86.9±4.2 | **96.2±0.7** |
| SR-MMP | 84.4±2.7 | 86.7±2.1 | 88.3±2.2 | 85.0±3.6 | 86.9±2.3 | **94.0±1.2** |
| SR-p53 | 83.3±4.6 | 84.2±12.7 | 83.4±4.0 | 84.0±5.2 | 86.7±3.5 | **95.4±1.0** |
| HIV | 92.2±1.3 | 92.3±2.5 | 89.0±2.5 | 83.5±2.8 | 87.6±2.2 | **97.2±0.2** |
| BACE | 81.7±2.5 | 78.6±8.7 | 81.0±2.1 | 77.5±5.4 | 80.0±1.9 | **80.5±5.0** |
| Regression tasks | | | | | | |
| Caco-2 | 0.370±0.025 | 0.362±0.053 | 0.322±0.024 | 0.325±0.020 | 0.319±0.022 | **0.311±0.075** |
| MDCK | 0.354±0.048 | 0.333±0.054 | 0.365±0.066 | 0.358±0.034 | 0.327±0.042 | **0.334±0.107** |
| PPB | 17.235±1.266 | 14.922±0.958 | 14.528±0.579 | 15.430±1.240 | 15.296±1.002 | **9.373±4.743** |
| VD | 0.927±0.127 | 1.264±0.390 | 1.076±0.108 | 1.036±0.284 | 1.014±0.131 | **0.773±0.253** |
| Fu | 0.456±0.018 | 0.466±0.015 | 0.508±0.402 | 0.437±0.022 | 0.421±0.016 | **0.356±0.057** |
| CL | 3.085±0.643 | 3.887±0.700 | 3.395±0.553 | 3.953±0.450 | 3.744±0.713 | **2.523±1.080** |
| Bioconcentration Factor | 0.898±0.063 | 0.777±0.097 | 0.840±0.104 | 0.673±0.081 | 0.711±0.076 | **0.553±0.091** |
| IGC50 | 0.703±0.043 | 0.481±0.085 | 0.461±0.042 | 0.420±0.033 | 0.448±0.028 | **0.377±0.033** |
| LC50FM | 1.026±0.106 | 0.944±0.098 | 0.904±0.075 | 0.921±0.085 | 0.889±0.129 | **0.745±0.118** |
| LC50DM | 1.280±0.106 | 1.171±0.114 | 1.206±0.129 | 1.277±0.165 | 1.136±0.176 | **0.786±0.210** |
| ESOL | 1.264±0.100 | 0.841±0.102 | 0.755±0.145 | 0.647±0.086 | 0.609±0.055 | **0.437±0.106** |
| FreeSolv | 2.576±0.648 | 1.255±0.478 | 1.125±0.154 | 0.991±0.186 | 1.123±0.104 | **0.950±0.539** |
| Lipophilicity | 0.829±0.035 | 0.669±0.068 | 0.653±0.048 | 0.587±0.036 | 0.695±0.042 | **0.446±0.061** |
| LogS | 1.253±0.046 | 0.868±0.053 | 0.841±0.047 | 0.802±0.049 | 0.843±0.030 | **0.682±0.177** |
| LogD | 0.697±0.026 | 0.604±0.028 | 0.499±0.017 | 0.447±0.018 | 0.568±0.020 | **0.394±0.048** |
| LogP | 0.830±0.012 | 0.436±0.020 | 0.398±0.020 | 0.367±0.024 | 0.518±0.011 | **0.338±0.070** |
